# Excellent color rendering index single system white light emitting carbon dots for next generation lighting devices

**DOI:** 10.1038/s41598-021-91074-w

**Published:** 2021-06-02

**Authors:** Manasa Perikala, Asha Bhardwaj

**Affiliations:** Department of Instrumentation and Applied Physics, Indian Institute of Science, Bangalore, 560012 India

**Keywords:** Engineering, Nanoscience and technology

## Abstract

Recently, quantum dots (QDs) are finding enormous application in white light emitting diodes (WLEDs) and WLEDs with high color rendition are in high demand. QD-WLEDs use different color (Red, Blue, Green) emitting QDs to obtain white light. Use of different color emitting QDs affect purity of white light due to self-absorption losses and QD degradation, in the long run affecting color rendering index (CRI) of WLEDs. Herein, we report low cost, environment friendly, open air atmosphere synthesis of single system white light emitting carbon dots (CDs) with broad emission bandwidth ranging 116 –143 nm and quantum yields (QY) ~ 5 – 13 % in colloidal state by modifying CD surface. Furthermore, carbon dot polymer phosphor (CD-PDMS phosphor) is fabricated which emits white light under UV illumination with a record emission bandwidth of ~ 154 nm and QY ~ 16 % in solid state. Moreover, CD-PDMS phosphor exhibit excellent color rendering index (CRI) ~ 96, the highest reported so far with CIE co-ordinates (0.31, 0.33) that are quite akin to pure white light. Such high performances are achieved due to high quality of CDs and CD-PDMS polymer phosphors by precise control in passivation/functionalization of nanoparticle surface. This work will set platform for the application of CD-phosphor based WLEDs in lighting systems.

## Introduction

White light emitting diodes are a major part of artificial lighting systems across the globe. Commercially available white LEDs use yellow emitting Cerium-doped Yttrium aluminium garnet (Ce: YAG) inorganic phosphor coated on blue emitting Indium Gallium Nitride (InGaN) chips^1,2^ or use RGB LEDs (RGB – Red + Blue + Green)^3,4^ to generate white light. Although WLEDs based on Ce: YAG yellow phosphor show minimum losses with high luminous efficacies, they exhibit poor red emitting spectral component in their emission spectra which decreases the CRI value of WLEDs^5,6^. Also, these phosphors are fabricated using highly expensive precursors at high reaction temperatures limiting their application in artificial lighting devices. Additionally, the emission bandwidth of these phosphors range from 20 to 60 nm^7^, such narrow emission bandwidth affects the quality of white light generated, further affecting the CRI of the WLED fabricated. CRI measures the ability of a light source to reveal the actual colors of objects as compared to ideal light source (sun light). High CRI is generally a desirable characteristic for an artificial lighting source to produce high quality white light. WLEDs with CRI > 80 are considered as good artificial lighting sources whereas WLEDs with CRI > 90 are considered as best sources for artificial lighting systems^8–12^. Although RGB based WLEDs have high CRIs, they tend to be more expensive alternatives with higher re-absorption losses affecting their luminous efficacies. Such inadequacies in lamp efficiency parameters paved the way for developing quantum dots (QDs) tri color phosphor materials with red, green and blue emitting dots coated on UV chip for WLED fabrication. So far, QD tri color phosphor based on CdSe-ZnSe QDs^13^, CdTe QDs^14^, CdSe/CdS/ZnS QDs, CuInS^15^ QDs, CuInS_2_/ZnS QDs^16^, InP/ZnS QDs^17^, CdSe/ZnS QDs^18^, Cu-doped ZnInS/ZnS QDs^19^ for WLED applications have been reported. Although QD based phosphors have many advantages in terms of luminous intensity, color tunability and energy consumption, most of these QD phosphors involve use of toxic materials such as Cd, Se, Te etc., which limit their applications. Also, WLEDs fabricated from these tri color QD phosphor suffer from degradation of QDs (that result from deterioration of material due to photo oxidation, heat or oxidation on exposure to open atmosphere or from self-aggregation) and re-absorption losses due to large size distribution. These re-absorption losses and degradation of QDs decrease CRI and overall device efficiency of QD based WLED.

Considering these shortcomings, there arises a high demand for the fabrication of broad emitting, highly stable, non-toxic, low cost QD phosphor using single white light emitting system, yielding high CRIs and device efficiencies with negligible re-absorption losses. Carbon dots (CDs), on the other hand are one of the most significant class of fluorescent carbon nano materials with least toxicity, stable high luminous intensities and broad emission spectra with outstanding resistance to photo bleaching and photo blinking^20–22^, making them excellent phosphor materials for WLEDs. Recently several reports have been published on the fabrication of carbon dot based WLEDs involving tri color WLEDs with CRI 92.9 and 81.1 with CIE color co-ordinate (0.3305, 0.3320) and (0.4046, 0.4028) respectively^23^, tri-color CD-WLED with CRI 92 and CIE (0.33, 0.34)^24^, orange emissive CD-WLED with CIE (0.41, 0.45)^25^, Orange emissive carbon dot phosphors for WLEDs with CRI 91 and CIE (0.41, 0.39)^26^, tri-CD films integrated with UV-LED chip based WLED with CRI of 96.5 and CIE (0.362, 0.370). These tri-color CD based WLEDs suffer from self-absorption losses and degradation of CDs with time, thus affecting CRI and quality of white light generated from these WLEDs.

In this paper we report in detail, one step fabrication of single system white light emitting CDs in open air atmosphere with quantum yields (QY) of 5 – 13 % in solution phase. Further, solid state white light emitting CD phosphor has been successfully fabricated and a QY of 16% is obtained. Furthermore, the fabricated CDs and CD polymer phosphor show broad emission bandwidth of ~ 116–154 nm, one of the highest reported so far for single white light emitting system^27,28^. This broad emission band leads to enhanced CRI of the phosphor^29^. The fabricated CD phosphor exhibited high quality white light with exceptional CRI ~ 96, the highest reported so far for single system white light emitting CD phosphor based WLED and CIE co-ordinates of (0.31, 0.33) that are akin to day light with CIE (0.32, 0.32). This work will set a platform for the fabrication of high CRI single system white light emitting carbon dot based WLEDs for artificial lighting systems.

## Experimental details

**Figure 1:**
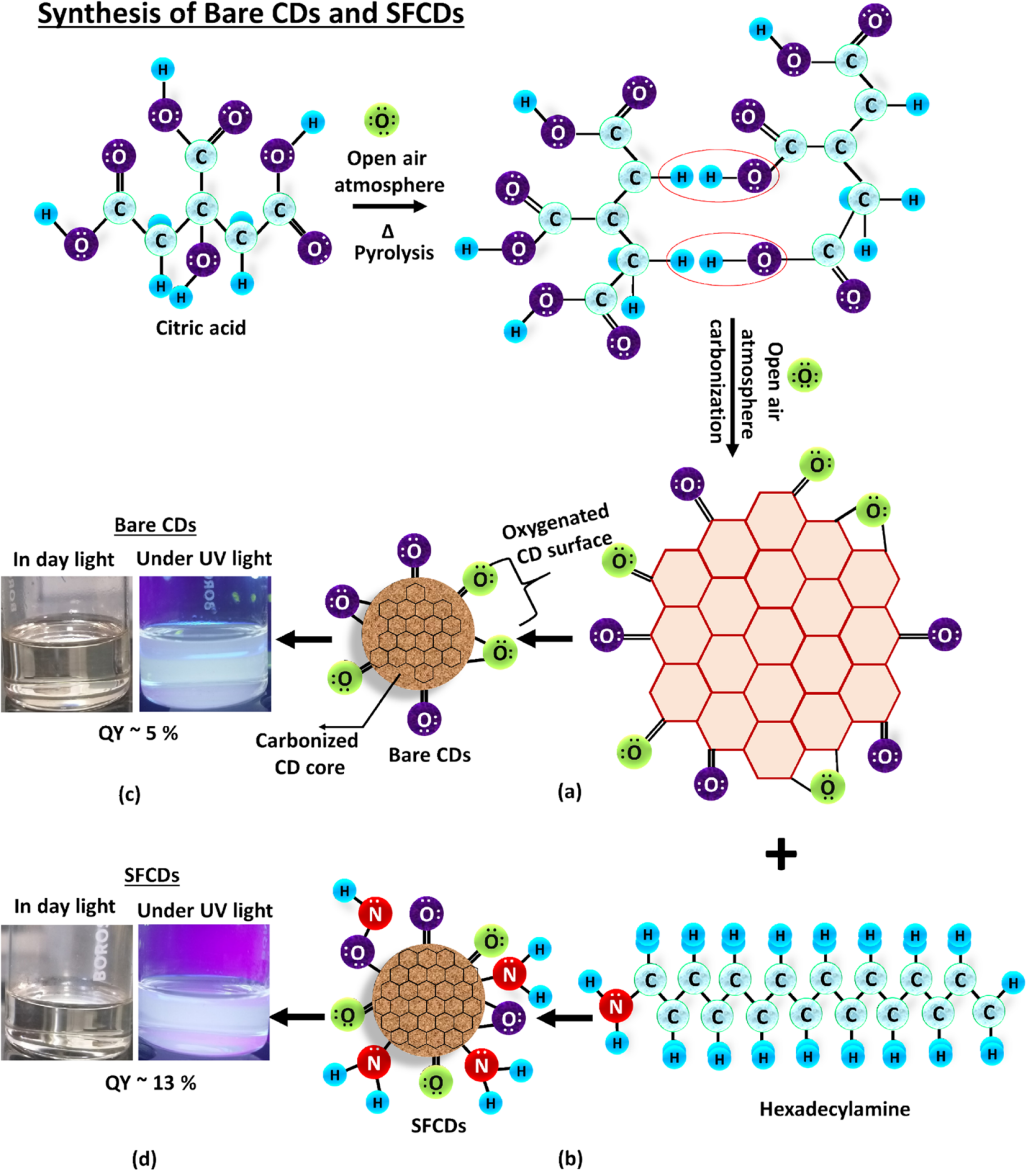
Schematic of synthesis of (**a**) bare CDs using citric acid as carbon precursor (**b**) SFCDs using citric acid as carbon precursor and hexadecylamine as surface functionalizing agent. Bare CD formation involves breakdown of citric acid molecules at higher temperatures accompanied by carbonization of carbon core. SFCDs precipitation involve breakdown of citric acid molecules at high temperatures (250 °C) accompanied by carbonization of carbon core followed by surface functionalization of CD surface with −NH_2_ groups from hexadecylamine. Open air atmosphere synthesis of CDs and SFCDs favour oxygenation of CD surface that involve oxygen containing groups getting attached to CD (and SFCDs) (such as C=O, C− O) surface which further act as emissive trap sites. These emissive sites excite at different energies enhancing the emission bandwidth of CDs (and SFCDs) which exhibit bright white light emission under UV illumination. (**c**) CDs and (**d**) SFCDs show white light emission under UV illumination.

Bare CDs are synthesized in open air atmosphere at temperatures as high as 250 °C using citric acid (1 g) as carbon precursor and octadecene (30 ml) as a non-coordinating solvent. Formation of bare CDs involve breakdown of citric acid molecules at higher temperatures accompanied by carbonization of carbon core (Fig. 1a). The fabricated CDs are purified using ethanol as a purifying agent and dispersed in chloroform for further analysis. Bare CDs have dangling bonds which leads to non-emissive trap states on the surface that decrease the luminescence intensity and QY of CDs. Henceforth, in order to enhance the emission characteristics and QY of CDs, CD surface is passivated using hexadecyl amine (HDA) as a surface functionalizing agent that provides −NH_2_ groups to CD surface (Fig. 1b). In order to check the affect of surface functionalization, SFCD samples with varied degree of surface functionalization are synthesized in open air atmosphere at 250 °C by maintaining constant amount of citric acid (1 g), octadecene (30 ml) and varying HDA concentration from 1.5 to 4 g in the reaction mixture. SFCD samples with HDA/ Citric acid molar concentration ratio ranging from 1.2 (1 g citric acid + 1.5 g HDA), to 3.5 (1 g citric acid + 4 g HDA) are synthesized. HDA/Citric acid molar concentration 1.2, 1.8 and 3.5 are labelled as SFCD_1.2_, SFCD_1.8_ and SFCD_3.5_ respectively throughout this report. Formation of SFCDs involve breakdown of citric acid molecules accompanied by carbonization of carbon core followed by surface functionalization of CD surface.

In order to enhance the stability of colloidal CDs and to ease their use in device applications, they are sealed in transparent polymer to protect them from environmental moisture^30^. Proper choice of polymer for packing CDs is made, such that it enhances the emission intensity, QY^31^ and quality of white light generated^32–38^. In this work CDs have been embedded in Polydimethylsiloxane (PDMS) polymer, considering its flexibility, high transparency, good dispersity^39–42^ and compatibility with CDs. For the fabrication of CD-PDMS polymer phosphor, PDMS and SYLGARD 184 curing agent are mixed in 10:1 ratio and stirred continuously for about ~ 5 min in a vial until the constituents get thoroughly mixed. The mixture is then degassed in a vacuum desiccator for about 15 min until a clear transparent mixture is observed. To this mixture 2 ml of fabricated CDs dispersed in chloroform are added and the entire solution is heated at 90 °C until chloroform evaporates from the mixture completely. The mixture is then transferred to a prefabricated plastic mold and allowed to rest at ambient conditions for about 48 h. After 48 h, the plastic mold is removed, and the fabricated CD polymer phosphor is ready for further characterization.

## Results and discussion

**Figure 2:**
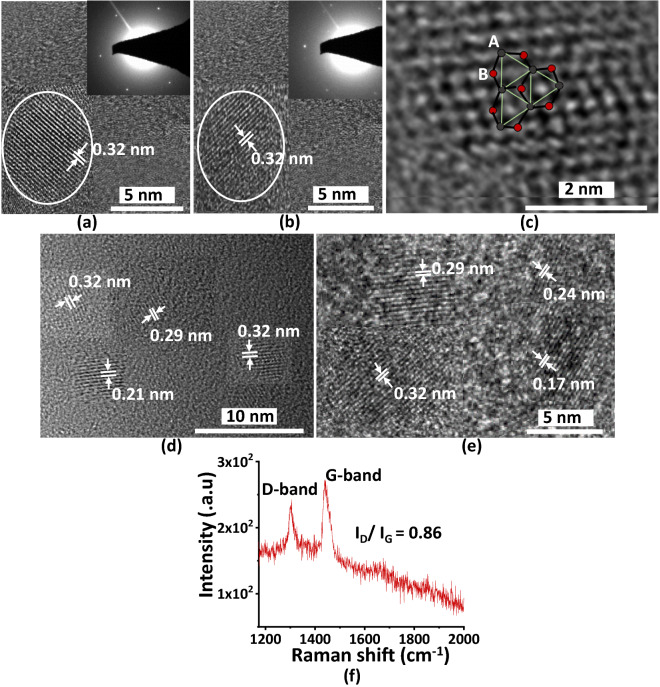
High Resolution TEM images of (**a**) bare CDs (**b**) SFCDs showing spherical CD formation with parallel lattice planes of d value ~ 0.32 nm that corresponds to (002) plane of graphitic crystal structure. SAED images of CDs and SFCDs (Inset of figure **a**,**b**) exhibit spot diffraction pattern supporting highly crystalline nature of fabricated CDs. (**c**) High magnification image of CDs showing hexagonal crystal structure. Additional HRTEM images of (**d**) bare CDs (**e**) SFCDs showing planar lattice fringes with d spacing of 0.17 nm, 0.21 nm, 0.29 nm and 0.32 nm that correspond to (004), (100), (101) and (002) planes of graphitic crystal structure (**f**) Raman spectra of bare CDs showing D and G bands of graphitic structure at 1302 cm^−1^ and 1441 cm^−1^ respectively. Further I_D_/I_G_ ratio in fabricated CDs is around ~ 0.86 supporting that the fabricated CDs have more ordered graphitic structure with less defects.

To explore the nanostructures, High Resolution Transmission Electron microscopy (HRTEM) and Selective Area Electron Diffraction (SAED) images are taken. HRTEM images confirm the formation of spherical CD nanoparticles (Figure S5) with prominent lattice fringes of 0.32 nm observed both in bare CDs (Fig. 2a) and SFCDs (Fig. 2b) that correspond to (002) lattice planes of graphitic crystal structure. SAED images of CDs and SFCDs (inset Fig. 2a,b) exhibit spot diffraction pattern supporting highly crystalline nature of fabricated CDs. High magnification image of CDs showing hexagonal crystal structure in the fabricated CDs (Fig. 2c). Additional HRTEM images of bare CDs (Fig. 2d) and SFCDs (Fig. 2e) showing planar lattice fringes with d spacing of 0.17 nm, 0.21 nm, 0.29 nm and 0.32 nm that correspond to (004), (100), (101) and (002) planes of graphitic crystal structure. Furthermore, the quality of graphitic structure in bare CDs is investigated by estimating the ordered and disordered phases using Raman spectra (Fig. 2f)^43,44^. Peaks at 1302 cm^−1^ and 1441 cm^−1^ correspond to the D and G bands of graphitic structure. D band represent defects (disordered *sp*^3^ hybridized carbon) and G band represents vibration of *sp*^2^ hybridized carbon atoms (ordered *sp*^2^ hybridized carbon) in graphitic structure. Intensity ratio i.e., I_D_/I_G_ represents graphitization in fabricated CDs. It is observed that I_D_/I_G_ ratio in fabricated CDs is ~ 0.86, which indicates that fabricated CDs have ordered graphitic structure with lesser number of defects. Obtaining Raman spectra for SFCDs was unsuccessful due to overlapping of strong fluorescence peaks with Raman signals.

**Figure 3:**
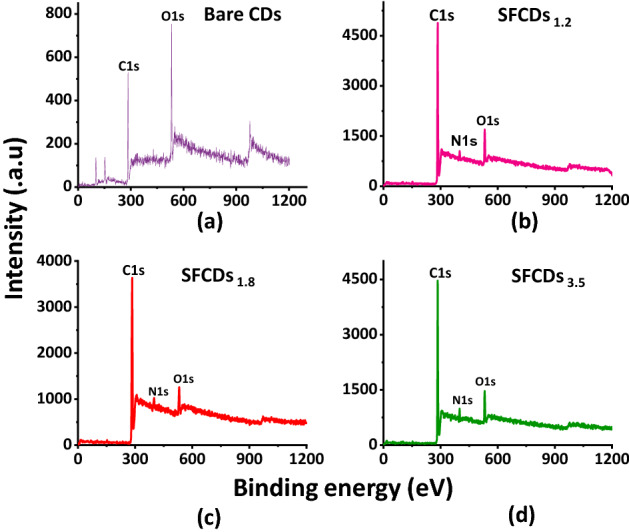
Full scan XPS spectra of (**a**) bare CDs (**b**) SFCD1.2 (**c**) SFCD1.8 (**d**) SFCD3.5, reveals C1*s* and O1*s* bands at 284.6 and 531.5 eV respectively. In addition, N1*s* band at 399.5 eV in SFCDs (**b**–**d**) indicate successful nitrogen doping in the fabricated SFCDs.

**Figure 4:**
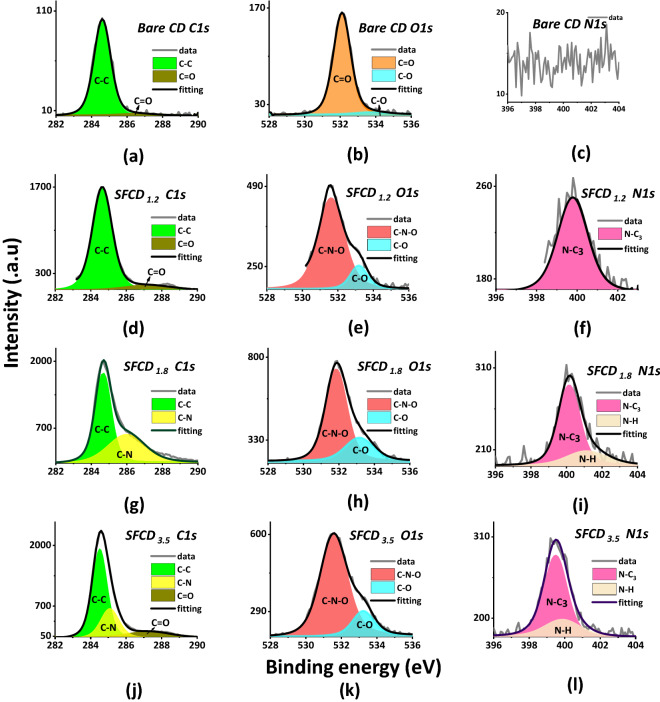
Further Deconvolution spectra of (**a**–**c**) C1*s*, O1*s*, N1*s* spectra of bare CDs, (**d**–**f**) C1*s*, O1*s*, N1*s* spectra of SFCD_1.2,_ (**g**–**i**) C1*s*, O1*s*, N1*s* spectra of SFCD_1.8_, (**j**–**l**) C1*s*, O1*s*, N1*s* spectra of SFCD_3.5_ is plotted. Deconvolution of high resolution C1s spectrum of bare CDs and SFCDs show two peaks at 284.6 eV and 287 eV that corresponds to C=C and C=O bonds respectively. Peak at 285 eV in C1s spectra of SFCDs correspond to C− N bonding. Further deconvolution of O1s spectrum of bare CDs resolve two peaks centered at 532 eV and 533 eV that corresponds to C=O and C− O groups whereas SFCDs show slightly different O1s spectra with peak centered at 531.5 eV corresponds to C− N−O bond. Furthermore, Deconvoluted N1s spectra of SCFDs show peaks centered at 400 eV and 401 eV that correspond to N−C_3_ and N−H bonds.

Furthermore, surface states of fabricated CDs are investigated using X-ray photo electron spectroscopy (XPS). XPS measurements elucidate the elemental composition and bonding in fabricated bare CDs and SFCDs. CDs and SFCDs are drop casted on a Si substrate in order to get their X-ray photoelectron spectra. Figure 3a–d depicts full scan XPS spectrum of bare CDs, SFCD_1.2_, SFCD_1.8_ and SFCD_3.5_. Wide scan spectrum (Fig. 3a–d) of bare CDs and SFCDs reveal C1s and O1s bands at binding energies of 284.6 eV and 531.5 eV respectively. Presence of N1s band at binding energy of 399.5 eV indicates successful nitrogen functionalization in the fabricated SFCD surface (Fig. 3b–d). Peaks at 101.6 and 152.5 eV are attributed to Si substrate. Furthermore, deconvolution of high resolution C1*s* spectrum of bare CDs and SFCDs show two peaks at 284.6 and 287 eV corresponding to C=C and C=O bonding respectively (Fig. 4a,d). In addition, peak at 285 eV in C1*s* spectra of SFCDs (Fig. 4g,j) correspond to C− N bonding. Deconvolution of O1*s* spectrum of bare CDs (Fig. 4b) resolve two peaks centered at 532 and 533 eV corresponding to C=O and C  O groups whereas SFCDs show slightly different O1s spectra (Fig. 4e,h,k) with peak centered at 531.5 eV corresponding to C− N−O bond. Furthermore, deconvoluted N1s spectra of bare CDs (Fig. 4c), SFCDs show peaks centered at 400 eV and 401 eV that correspond to N− C_3_ and N−H bonds (Fig. 4f,i,l). Presence of N−H groups in N1s spectra of SFCDs and absence of these groups in bare CDs support surface functionalization of SFCDs by amino groups in HDA. It is observed that surface functionalization of CD surface increases the carbonization of carbon core supported by increase in C=C peak intensities of SFCDs compared to bare CDs. Also, Surface functionalization of CD surface increases the number of surface states on CD surface supported by increase in C−O, N−H and C=O peak intensities of SFCDs as compared to bare CDs respectively. Further, percentage of carbon and nitrogen in SFCDs as analyzed from XPS spectra amounts to ~ 64% and ~ 14% respectively indicating the fabricated SFCDs are nitrogen functionalized and carbon rich.

**Figure 5:**
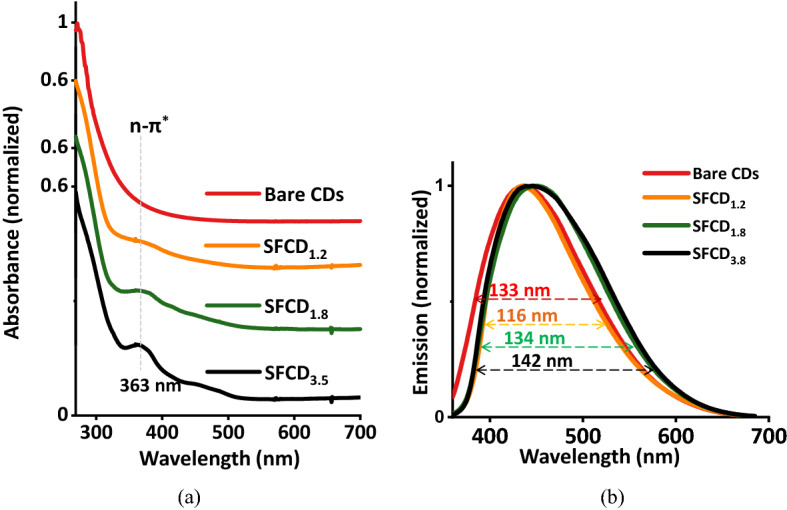
(**a**) Absorption and (**b**) emission spectra of colloidal CDs and SFCDs. Bare CDs show a broad absorption window ranging from 270 to 400 nm. This broad absorption spectra is a cumulative effect of electronic transitions between intrinsic carbon domains (π-π* electronic transitions) and between oxygenated surface groups on CD surface with intrinsic carbon domains (n-π* electronic transitions). SFCDs exhibit an absorption peak at 363 nm which intensifies and becomes prominent with increase in degree of surface functionalization from SFCD_1.2_ to SFCD_3.5_. This peak corresponds to electronic transitions (n-π* electronic transitions) between surface groups (− NH_2_ created by HDA) with intrinsic carbon domain. Further, bare CDs excited at 350 nm excitation wavelength, exhibit a broad emission spectrum ranging from 360 to 700 nm with a bandwidth of 133 nm which is highly expected for white light emitting sources. SFCD samples show bandwidth of 116 nm (SFCD_1.2_), 134 nm (SFCD_1.8_) and 143 nm (SFCD_3.5_) with increase in degree of surface functionalization.

In order to further investigate the CDs and SFCDs, their emission and absorption characteristics are studied. Absorption spectra of the CDs were recorded from 269 to 700 nm range. Bare CDs exhibit a broad absorption window ranging from 270 to 400 nm as observed in the absorption spectra (Fig. 5a). This broad absorption spectrum is a cumulative effect of electronic transitions between intrinsic carbon domains (π-π* electronic transitions) and between oxygenated surface groups on CD surface with intrinsic carbon domains (n-π* electronic transitions). Open air atmosphere synthesis of CDs favour oxygenation of CD surface that involve oxygen containing groups getting attached to CD surface supported by XPS and FTIR analysis. These oxygen containing groups (such as C=O, C−O) act as emissive trap sites enhancing the luminescent properties of these dots. However, SFCDs absorb from 270 to 500 nm, exhibiting an absorption peak at 363 nm which intensifies and becomes prominent with increase in degree of surface functionalization from SFCD_1.2_ to SFCD_3.5_. This peak corresponds to electronic transitions (n-π* electronic transitions) between surface groups (−NH_2_ created by HDA) and intrinsic carbon domain. In order to further investigate the nanoparticles, emission characteristics of bare CDs and SFCDs are studied. As fabricated nanoparticles emit white light when exposed to UV lamp (peak wavelength of 350 nm) (Fig. 1c,d). Considering this, bare CDs are excited at 350 nm excitation wavelength and emission spectra recorded. CDs exhibit broad emission spectrum (Fig. 5b) ranging from 360 to 700 nm with a bandwidth of 133 nm which is expected for white light emitting sources. SFCDs emit from 360 to 700 nm on excitation with 350 nm source. Emission bandwidth changes from 116 to 143 nm with increase in degree of surface functionalization from SFCD_1.2_ to SFCD_3.5_. Both bare CDs and SFCDs show broad emission spectra due to the presence of different surface groups (C=O, C−O, N−H) which excite at different energies and emit correspondingly that favours bright white light emission under UV illumination (Fig. 1c,d). Emission spectra of SFCD_3.5_ is red shifted by 10 nm when compared to bare CDs (Fig. 5b). The red shift is accounted to surface states (surface oxygenation, surface functionalization) and size (carbonization) effects. Increase in degree of surface functionalization of CD surface leads to an increase in electron cloud around CD which increases the electronic conjugation of lone pair of electrons from the surface groups with π orbitals of carbon core. Increased electronic conjugation of lone pair of electrons from the surface groups with π orbitals of carbon core decreases the energy gap between n (state due to surface groups) and π^*^ (carbon core anti bonding orbital) electronic states. Additionally, in SFCDs, increased degree of carbonization of CD leads to increase in electron cloud in CD core that increases the electronic conjugation of π–π^*^ orbitals of carbon core. Increase in electronic conjugation of π–π^*^ orbitals of carbon core decrease the energy gap between π–π^*^ electronic transitions. Thus, the red shift is attributed to a cumulative effect of functionalization and carbonization in SFCDs.

**Figure 6:**
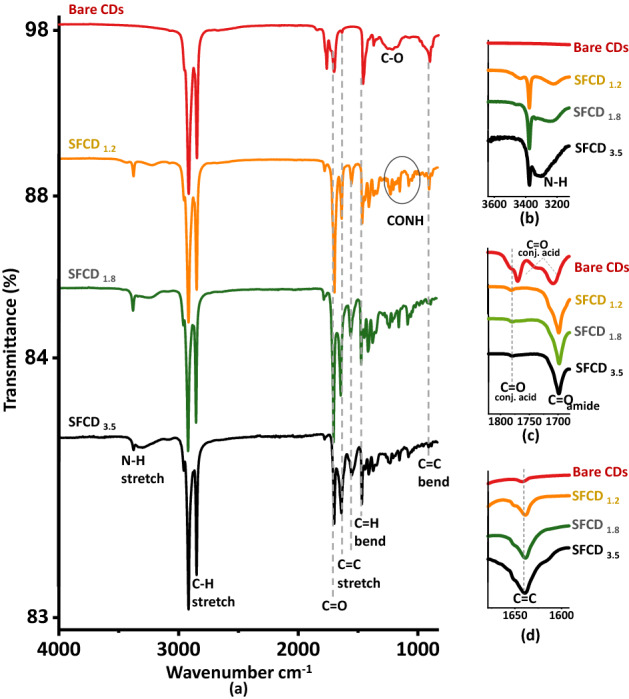
(**a**) FTIR spectra of bare CDs and SFCDs. Bare CDs FTIR spectra show C=O stretching vibrations of conjugated acid at 1779 cm^−1^, 1768 cm^−1^ and 1708 cm^−1^, C=C stretching vibration at 1638 cm^−1^, and a broad band of C−O stretching vibrations at 1218 cm^−1^. SFCDs show signature peak of N−H stretching vibrations at ~ 3436−3218 cm^−1^, C=O stretching vibration of primary amide at 1698 cm^−1^ and a band of CONH stretching vibrations at 1233 cm^−1^. Peaks at 2918, 2847 cm^−1^ correspond to C−H stretching vibrations and peaks at 1468 cm^−1^, 908 cm^−1^ correspond to C−H bending and C=C bending modes respectively. Further figure shows enlarged image of (**b**) N−H stretch region, (**c**) C=O stretch region and (**d**) C=C stretch region. It is observed that with increase in surface functionalization (SFCD_1.2_ to SFCD_3.5_) an increase in the peak width and intensity of N−H stretching vibrations and an increase in the peak intensities of C=O, C=C stretching vibrations are observed.

In order to have more insight into the functional groups attached to CD surface and the effect of degree of surface functionalization on carbonization of carbon core and surface oxygenation of CD surface, Fourier Transform Infrared Spectroscopy of bare CDs and SFCDs (Fig. 6a) is recorded. FTIR spectra of bare CDs reveal characteristic peaks of C=O stretching vibrations of conjugated acid at 1779 cm^−1^, 1768 cm^−1^ and 1708 cm^−1^, C=C stretching vibration at 1638 cm^−1^ and a broad band of C−O stretching vibrations at 1218 cm^−1^ which support that bare CD surface is embellished with C=O and C−O surface groups as indicated by XPS analyses. SFCDs show signature peak of N−H stretching vibrations at ~ 3436 to 3218 cm^−1^, C=O stretching vibration of primary amide at 1698 cm^−1^ and a band of CONH stretching vibrations at 1233 cm^−1^. With increase in surface functionalization (SFCD_1.2_ to SFCD_3.5_) an increase in the peak width and intensity of N−H stretching vibrations (Fig. 6b) and an increase in the peak intensity of C=O stretching vibrations (Fig. 6c) is observed. This increase in peak intensities upon surface functionalization support increase in number of emissive sites as observed in absorption spectra of SFCDs (also observed in XPS analyses). Peaks observed in bare CDs and SFCDs at 2918 cm^−1^, 2847 cm^−1^ correspond to C−H stretching vibrations and peaks at 1468 cm^−1^, 908 cm^−1^ correspond to C−H bending and C=C bending modes respectively. However, in SFCDs an increase in the intensity of C=C stretching vibrations is observed compared to bare CDs which support an increase in degree of carbonization of carbon core upon surface functionalization (Fig. 6d). Henceforth, functionalization of CD surface increases the number of surface emissive states on CD surface and intrinsic carbon emissive states in CD core which further enhances the quality of CD emitting white light.

Further Quantum yield measurements of the fabricated CDs and SFCDs are taken to check the quality of fabricated CDs. It is observed that bare CDs exhibit a QY of ~ 5% and SFCD_1.2_ exhibit a QY of ~ 8%, SFCD_1.8_ exhibit a QY of ~ 12% and SFCD_3.5_ exhibit a QY of ~ 13%. Surface functionalization of CD surfaces passivates the non-emissive surface states and increases the number of emissive groups (such as −NH_2_, C=O) on CD surface, also an increase in number of C=C intrinsic carbon domains (supported by FTIR) which further enhances the QY of CDs.

**Figure 7:**
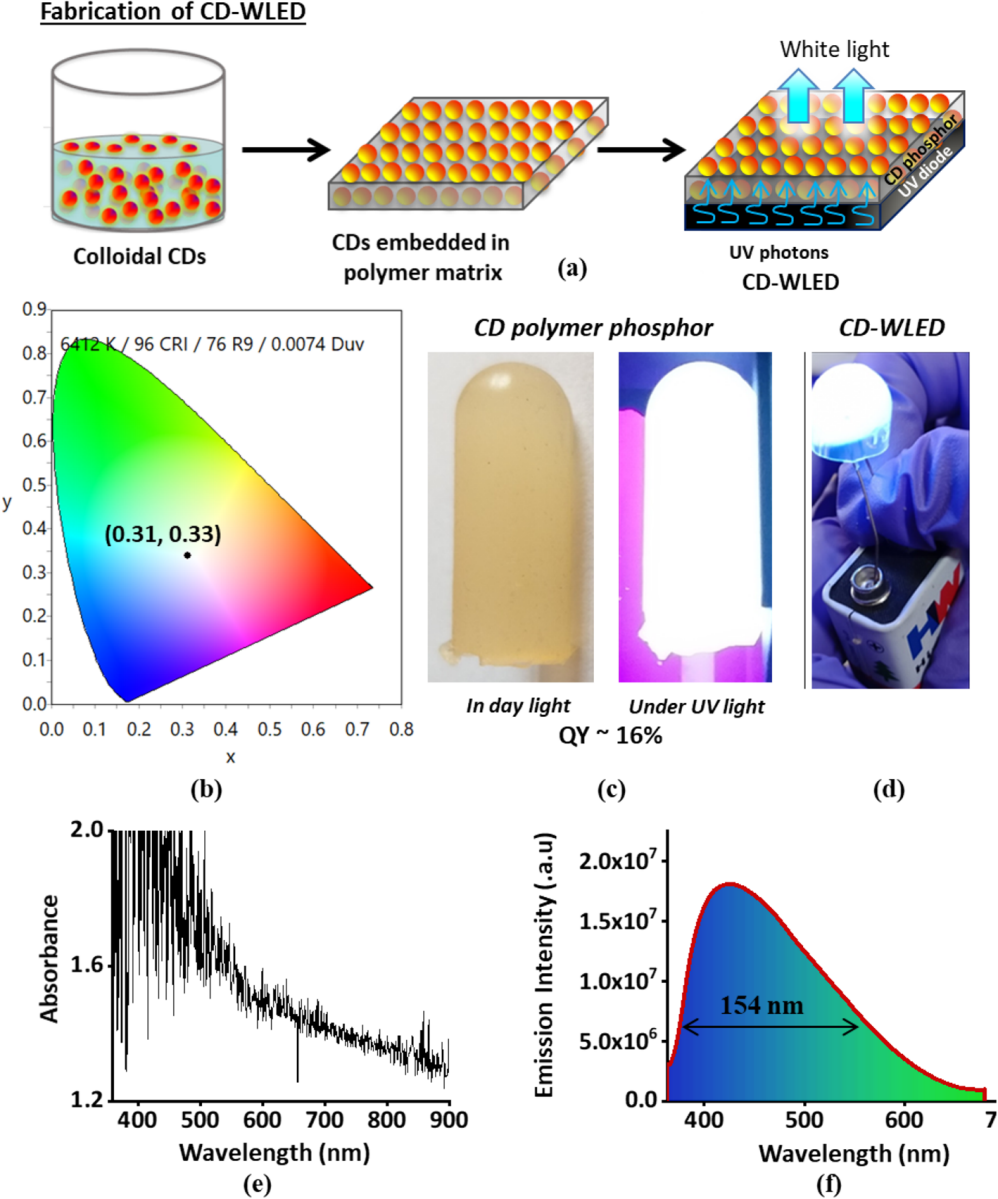
(**a**) Schematic of fabrication of CD-WLED. (**b**) Shows CIE color co-ordinates of fabricated CD polymer phosphor. White light emitted from CD phosphor show CIE chromatic co-ordinates of (0.31, 0.33) and a CRI of ~ 96 making it as an excellent phosphor for lighting systems. (**c**) CD polymer under visible light and under UV light illumination (**d**) Preliminary demonstration of polymer coated UV-LED (fabricated by us in our lab) which acts as white light emitting diode (WLED) (**e**) Absorption and (**f**) Emission spectra of CD polymer phosphor. CD polymer phosphor exhibits a broad absorption band ranged at ~ 350−700 nm. Under 350 nm excitation, CD polymer phosphor produce very broad white emission band ranging from 360 to 700 nm with a bandwidth of ~ 154 nm that favors bright white light emission.

In order to further enhance the application of these white light emitting colloidal nanomaterials, it is vital to embed them in a solid material. PDMS is the choice of solid material because of its good flexibility, good dispersity, compatibility with CDs and transparency to visible light. CDs embedded in PDMS have been fabricated and analysed for their optical properties (Fig. 7a). To investigate the luminescent behavior of CD polymer phosphor, absorption and emission spectra is recorded as shown in (Fig. 7e,f). The CD polymer phosphor shows a broad absorption band ranging from ~ 350 to 700 nm. Under 350 nm excitation, CD polymer phosphor produce very broad white emission band ranging from 360 to 700 nm with a bandwidth of ~ 154 nm (Fig. 7f). This CD polymer phosphor emits bright white light under UV illumination (Fig. 7c) with a quantum yield of ~ 16%. This enhancement in QY in CD-PDMS polymer compared to colloidal CDs (QY of 5%) is due to effective passivation of dangling bonds and non-emissive trap sites on CD surface by PDMS polymer matrix (for FTIR of CD-PDMS polymer phosphor please refer supplementary data (Figure S4). Further, the quality of white light generated, and the efficiency parameters of the fabricated CD phosphor are examined as shown in Fig. 7b. It is observed that white light emitted from CD polymer phosphor show Commission Internationale de l’Eclairage (CIE) chromatic co-ordinates of (0.31, 0.33) which are very close to day light from sun with CIE chromatic co-ordinates of (0.32, 0.32), indicating that CD phosphor emit high quality pure white light. The emitted broad band white light emission of ~ 154 nm bandwidth exhibit a color rendering index (CRI) of ~ 96 the highest reported so far for single system white light emitting CD phosphor based WLED, making it an excellent phosphor for lighting systems that require very accurate color discrimination (An ideal artificial lighting device is supposed to have CRI > 80). Application of CD polymer phosphor has been tested and demonstrated by coating it on a UV LED (Fig. 7d). It is observed that CD polymer coated LEDs emit excellent bright white light. The demonstration successfully indicates that this CD polymer probably can be used in WLEDs. A detailed study on the important optical parameters (such as CRI, CIE, efficiency) of CD polymer based WLEDs will be a subject of further research.

## Conclusions

In summary we report open air atmosphere synthesis of highly luminescent, single system white light emitting carbon dots and carbon dot phosphor for next generation white light emitting devices. Fabricated CDs emit white light with a Quantum yield of 5–13% in colloidal state by modifying the surface of CD fabricated. Further material and optical characterizations of the fabricated CDs reveal the effect of carbonization, oxygenation and surface modifying on the quality of white light emission from these dots. Furthermore, fabricated CD-PDMS polymer phosphor at 350 nm excitation show highly intense bright white light emission with a QY of ~ 16. White light emitted from CD polymer phosphor show chromatic color co-ordinates of (0.31, 0.33) with a color rendering index of ~ 96, the highest reported so far for single system white light emitting CD phosphor based WLED supporting its highest color purity, and quality of white light generated. Henceforth, the fabricated CD polymer phosphor finds its highest potential in fabricating eco-friendly high quality white light emitting devices.

## Supplementary Information

Supplementary Information.
