# Supplementary material for: Excellent color rendering index single system white light emitting carbon dots for next generation lighting devices
Source: Sci Rep. 2021 Jun 2;11:11594. doi: 10.1038/s41598-021-91074-w (PMC8172867; doi:10.1038/s41598-021-91074-w)
Supplement: Supplementary file 1 — Supplementary Information. [file 41598_2021_91074_MOESM1_ESM.docx]

**Supplementary information:**

Additional information depicting figures supporting TEM, XRD of Bare CDs and SFCDs, Comparison between emission spectra of Bare CDs and SFCDs synthesized in open and inert atmospheres, Emission spectra of CD-PDMS phosphor at different excitation energies, FTIR of CD-PDMS polymer phosphor are discussed.

**XRD traces of bare CDs and SFCDs**

|  |
| --- |
| Figure S1: XRD traces of synthesized bare CDS, SFCD_1.2_, SFCD_1.8_, SFCD_3.5_ supporting crystalline nature in the fabricated CDs. Broad diffraction peak at 19-21^ο^ is observed in bare CDs that corresponds to (002) plane of the graphitic crystal structure. With increase in extent of surface functionalization (increase in HDA concentration) the diffraction peak gets narrow supporting increase in crystallinity in the fabricated SFCDs. |

**Comparison between emission spectra of Bare CDs and SFCDs synthesized in open and inert atmospheres**

|  | | |
| --- | --- | --- |
| (a) | (b) | (c) |
| Figure S2: Emission spectra of (a) bare CDs (b) SFCDs synthesized in open and inert atmospheres. (c) shows emission of CDs and SFCDs fabricated, under UV illumination. Although bare CDs synthesized in open and inert atmospheres emit white light under UV-illumination, emission band width and Quantum yield of bare CDs synthesized in open atmosphere is higher (emission bandwidth-133nm, QY-5%) compared to that of bare CDs synthesized in inert atmosphere (emission bandwidth-122nm, QY-4%). This is due to surface oxygenation of CDs increasing the number of emissive states in open atmospheres. SFCDs synthesized under inert atmosphere show green light emission under UV-illumination with emission bandwidth of 166nm and QY of 31% whereas SFCDs synthesized in open atmospheres show bright white light emission under UV-illumination with emission bandwidth of 135 nm and QY of 13%. | | |

**Emission spectra of CD-PDMS phosphor at different excitation energies**

|  | |
| --- | --- |
| (a) | (b) |
| Figure S3: (a) un normalized (b) normalized PL spectra of fabricated CD-PDMS polymer phosphor at different excitation wavelengths (λ_ex_) ranging from 250-450 nm. An increase in emission intensity is observed with increase in λ_ex_ from 250 to 380 nm. On further increase in λ_ex_ from 380-450 nm, a decrease in emission intensity is observed. Further a red shift of ~ 109 nm in peak/max emission wavelength is observed with increase in λ_ex_ from 250 to 450 nm. This is due to the varied emissive states present on the CD surface exciting at different energies and emitting correspondingly along with varied particle size distribution in the synthesized CDs. | |

**FTIR of CD-PDMS polymer phosphor**

|  | |
| --- | --- |
| (a) | (b) |
| Figure S4: FTIR spectra of (a) Bare CDs, (b) CD-PDMS polymer phosphor. Peaks at 1735, 1411 and 2965 cm^-1^ correspond to C=O, C=C and C-H stretching vibrations respectively. It is observed that few groups that were initially present on Bare CDs (C=O, C-O)have lesser intensity compared to that in CD-PDMS phosphor indicating that the surface in CD-PDMS polymer phosphor is more passivated as compared to colloidal CDs. | |

**Tem image of CDs showing size distribution**

| 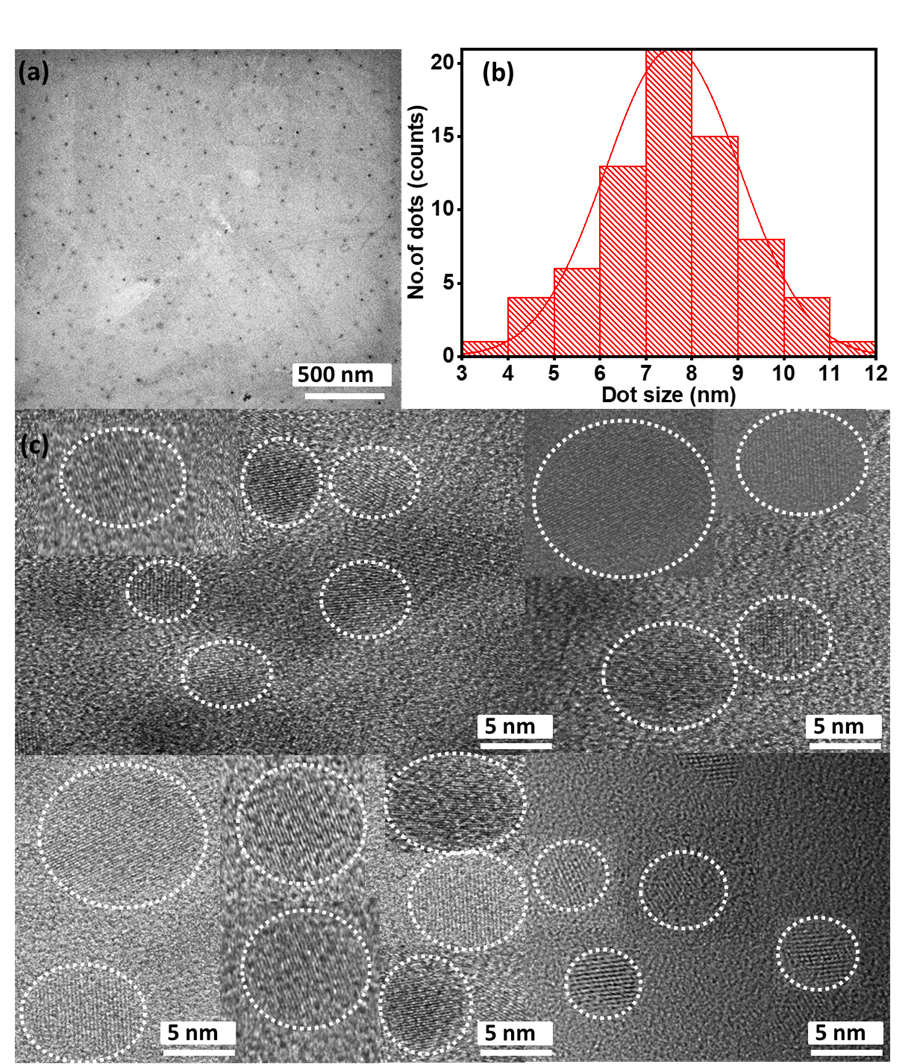 |
| --- |
| Figure S5: (a, c) TEM images of bare CDs at different magnifications. (b) Considering the varied sizes of particles obtained from TEM images a histogram is plotted showing the average size of CD formed ~8 nm. |
